# Knowledge Mapping of Macrophages in Osteoporosis: A Bibliometric Analysis (1999–2023)

**DOI:** 10.1111/os.14159

**Published:** 2024-07-09

**Authors:** Hai‐Feng Jia, Han‐Zheng Li, Yi‐Fa Rong, Kai Jiang, Xue‐Zhen Liang, Gang Li

**Affiliations:** ^1^ First College of Clinical Medicine Shandong University of Traditional Chinese Medicine Jinan China; ^2^ Orthopaedic Microsurgery Affiliated Hospital of Shandong University of Traditional Chinese Medicine Jinan China

**Keywords:** Bibliometrics, Exosome, Macrophages, mTOR, Osteoporosis, VOSviewer, Web of Science (WoS)

## Abstract

**Background:**

Osteoporosis is a common metabolic disorder that significantly impacts quality of life in the elderly population. Macrophages play a crucial role in the development of osteoporosis by regulating bone metabolism through cytokine secretion. However, there is a lack of scholarly literature in the field of bibliometrics on this topic.

**Objective:**

This study provides a detailed analysis of the research focus and knowledge structure of macrophage studies in osteoporosis using bibliometrics.

**Methods:**

The scientific literature on macrophage research in the context of osteoporosis, retrieved from the Web of Science Core Collection (WoSCC) database spanning from January 1999 to December 2023, has been incorporated for bibliometric examination. The data is methodically analyzed and visually represented using analytical and visualization tools including VOSviewer, CiteSpace, Scimago Graphica, the Bibliometrix R package, and Pajek.

**Results and Conclusions:**

In the last quarter‐century, there has been a consistent rise in the quantity of scholarly publications focusing on the relationship between macrophages and osteoporosis, resulting in a total of 1499 research documents. These studies have originated from 45 different countries, with China, South Korea, and the United States being the most prominent contributors, and the United States having the highest frequency of citations. Noteworthy research institutions involved in this field include Shanghai Jiao Tong University, Wonkwang University, Huazhong University of Science and Technology, and Seoul National University. The *Journal of Bone and Mineral Research* is widely regarded as the premier and most frequently referenced publication in the field. These publications involve the collaboration of 8744 authors, with Lee Myeung Su contributing the most articles, and Takayanagi being the most co‐cited author. Key emerging research focal points are encapsulated in keywords such as “mTOR,” “BMSCs,” “bone regeneration,” and “exosome.” The relationships between exosome from macrophage sources and those from BMSCs, along with the regulatory role of the mTOR signaling pathway on macrophages, represent crucial directions for future development in this field. This study represents the inaugural comprehensive bibliometric analysis detailing trends and advancements in macrophage research within the osteoporosis domain. It delineates recent frontiers and hotspots, providing valuable insights for researchers in this particular area of study.

## Graphical abstract

Retrieve literature from the WOS Core Collection (WOSCC) using specific selection criteria, and employ bibliometric tools to analyze and generate a knowledge graph.

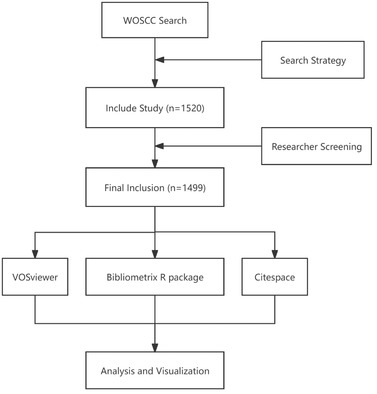

## Introduction

Osteoporosis (OP) is a skeletal metabolic disorder marked by a systemic decline in bone mass and heightened bone fragility, resulting in bone loss and microstructural deterioration.^1^ Initial stages of the disease may be asymptomatic, but as it advances, patients may suffer from pain, skeletal deformities, and, in severe instances, osteoporotic fractures, leading to significant economic implications for individuals and society. The worldwide prevalence of OP is estimated to be around 19.7%, with a notable 40.4% risk of decreased bone mass.^2^ In China, data^3^ shows a prevalence of OP of 32% among individuals aged 65 and older, with rates of 10.7% in males and 51.6% in females. U.S. health reports^4^ indicate that over 10 million individuals aged 50 and older are affected by OP, and more than 34 million are at risk of developing the condition. The rising fragility of bones is a contributing factor to the increased occurrence of fractures. Worldwide, approximately one‐third of females and one‐fifth of males aged 50 and above suffer from osteoporotic fractures.^5^ Vertebral fractures, hip fractures, and distal forearm fractures are prevalent occurrences, exerting substantial pressure on healthcare expenditures in various regions globally.^6^ As the aging of the global population intensifies, the threat posed by OP to human health becomes increasingly pronounced. In recent years, there has been a widespread utilization of whole‐genome association studies and various omics technologies, such as genomics, transcriptomics, epigenomics, proteomics, and metabolomics, in the exploration of osteoporosis mechanisms.^7^ Nevertheless, due to the complexities inherent in biological processes, a thorough understanding of the pathological landscape of osteoporosis remains incomplete. Therefore, the urgent and unresolved imperative of elucidating the etiology and pathogenic mechanisms of osteoporosis to facilitate early intervention strategies for prevention persists.

Derived from hematopoietic stem cells within the bone marrow, macrophages are a component of the mononuclear phagocyte system, distributed throughout the majority of bodily tissues. These cells play a crucial role in diverse biological functions such as bacterial phagocytosis, inflammatory reactions, antigen processing and presentation, and immune modulation.^8^ Research^9^ has demonstrated a notable association between macrophages and the development and advancement of osteoporosis. The regulation of bone resorption and formation in the human skeleton is predominantly controlled by osteoblasts and osteoclasts, with macrophages playing a significant role in the differentiation of osteoclasts. The interaction between macrophages and osteoclasts, known as the “macrophage‐osteoclast axis,” is crucial in maintaining the equilibrium between bone remodeling and absorption.^10^ Upon activation by the receptor activator of nuclear factor kappa‐B ligand (RANKL), macrophages undergo differentiation into osteoclasts, facilitating the process of bone resorption.^11^ Moreover, resident bone macrophages play a role in bone formation through the clearance of apoptotic bone cells and the facilitation of mineral deposition by osteoblasts.^12^ Additionally, primary macrophages have the ability to differentiate into M1 and M2 phenotypes *via* distinct pathways, displaying pro‐inflammatory and anti‐inflammatory properties,^13^ respectively. These cells are involved in the regulation of inflammatory factor secretion and contribute to bone remodeling and repair mechanisms.^14^, ^15^, ^16^ Although numerous studies focus on macrophages in the field of osteoporosis, comprehensive and high‐quality reviews are often lacking, a gap that bibliometric analysis can effectively address. Bibliometrics has become a valuable tool for summarizing the knowledge structure and forecasting future trends within a specific field through the analysis of scientific literature.^17^ Its significance is evident in research pertaining to various diseases, including cancer,^18^ Cardiorenal Syndrome,^19^ and rheumatoid arthritis.^20^

To gain further insights into the current status and future trends of macrophage research in the field of osteoporosis, this study employs bibliometric methods to qualitatively and quantitatively analyze the knowledge structure and research focal points related to the pathogenesis, diagnosis, and treatment of osteoporotic diseases involving macrophages over the past 25 years. Software tools like VOSviewer, CiteSpace, Scimago Graphica, Bibliometrix R‐package, and Pajek offer robust data analysis and visualization capabilities that are commonly utilized in bibliometric research. This research employs various software systems to analyze annual publication volumes, countries and institutions, key authors, major contributing journals, core literature, keywords, and other relevant factors, with the objective of comprehending developmental trends and research frontiers. The results are intended to provide a valuable resource for scholars in the respective field.

## Sources and Methods

### Data Sources

**FIGURE 1:**
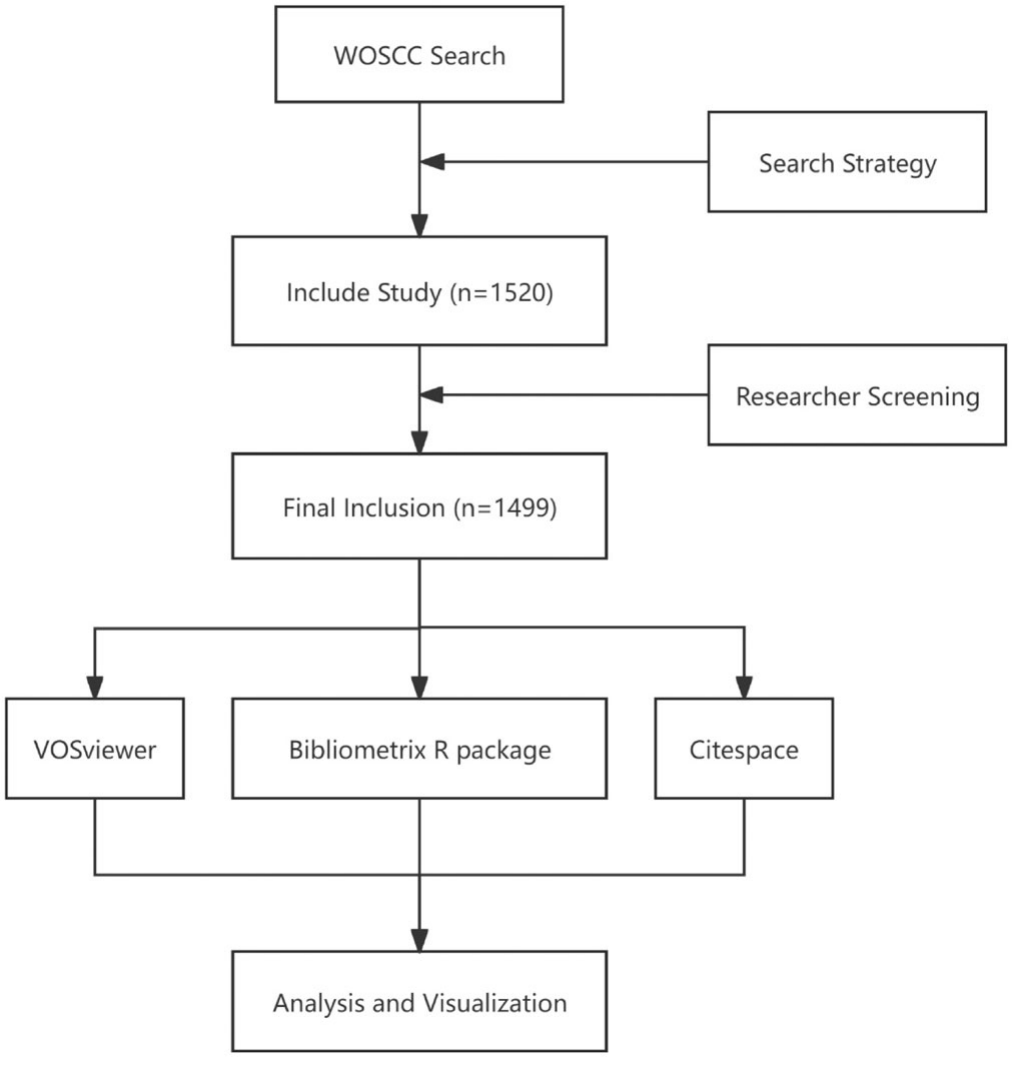
Publications screening flowchart.

Literature retrieval was conducted in the Web of Science Core Collection (WoSCC) database (https://www.Webofscience.com/wos/woscc/basic-search) based on the following inclusion criteria: (i) the search query was set as (TS = (macrophage)) AND TS = (osteoporosis); (ii) the search period ranged from January 1, 1999, to December 31, 2023; (iii) language was limited to English, and document types were specified as “articles” and “review.” A total of 1520 documents were initially identified. The exclusion criteria were applied as follows: (i) documents that were not peer‐reviewed, such as editorials and conference abstracts, were excluded; (ii) studies that did not focus explicitly on the role of macrophages in osteoporosis were excluded; (iii) articles that were retracted or contained incomplete data were also excluded. Following these criteria, 1499 documents were deemed suitable for inclusion in the study. Complete records of the included literature were downloaded, encompassing titles, abstracts, keywords, publication years, authors, nationalities, journal names, research directions, publishing institutions, funding agencies, and references. All data were downloaded on the same day, January 17, 2024 (Figure 1).

### Research Methods

Statistical analysis of annual publication counts was quantitatively analyzed using the statistical analysis functionality of the WoSCC database and visualized through Excel 2021. In this study, the widely utilized literature analysis and visualization tools, VOSviewer and CiteSpace, were employed. VOSviewer, which operates on the principles of co‐citation and co‐citation network analysis, offers a comprehensive bibliometric knowledge map from diverse viewpoints.^21^ The analysis of institutions, journals, authors, citations, and keywords in this study utilized VOSviewer (version 1.6.20, https://www.vosviewer.com/). Additionally, Scimago Graphica software^22^ (Beta version 1.0.38, www.graphica.app) was employed in conjunction with VOSviewer to visualize the national publication output on a map. Pajek (version 5.18, http://mrvar.fdv.uni-lj.si/pajek/) is frequently utilized for analyzing intricate nonlinear networks,^23^ and in conjunction with VOSviewer, it offers an enhanced visualization of author keywords.

Citespace (version: 6.2.R7, https://citespace.podia.com/), developed by Professor Chaomei Chen at Drexel University in the United States, is a software used for the quantitative analysis of scientific literature data.^24^ This study utilizes co‐citation analysis theory and pathfinding network algorithms to efficiently elucidate the evolution of a particular research trajectory. Through visual mapping, it emphasizes significant literature and research groups. Citespace was predominantly employed in this research for the creation of burst citation maps.

The Bibliometrix R package (https://www.bibliometrix.org) conducted comprehensive bibliometric analysis from multiple dimensions,^25^ facilitating literature clustering to identify disciplinary hotspots and development trends. In this study, Bibliometrix R package (version 4.0) was utilized for analyzing the research trends of author keywords.

## Result

### Annual Quantitative Analysis of Publications

**FIGURE 2:**
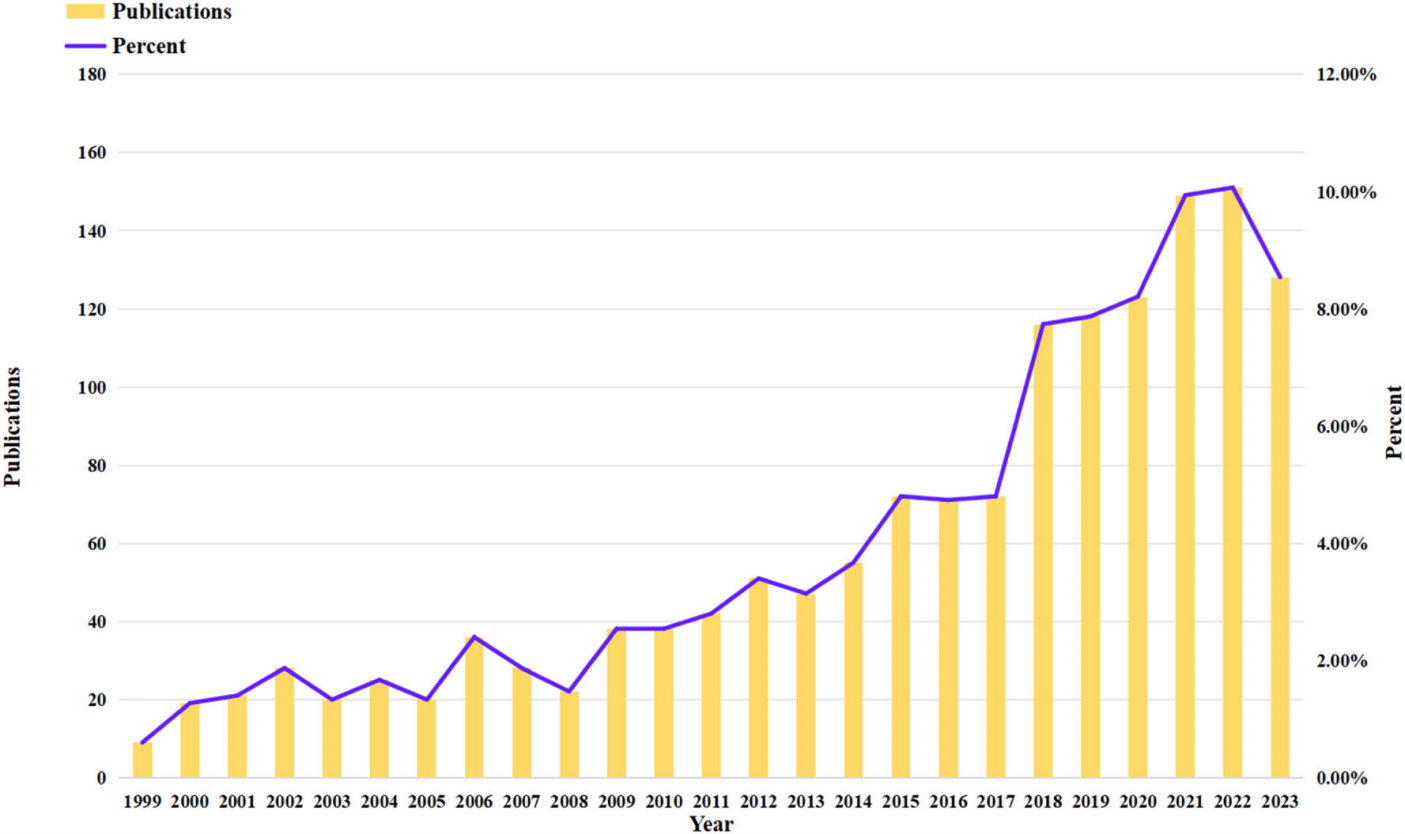
Annual output of research of macrophages in OP.

The quantity of articles published during a specified timeframe serves as a key indicator of research activity and advancement within a specific field, offering insights into the theoretical sophistication and pace of growth in that area of study. The findings of the study indicate a consistent upward trend in research on osteoporosis and macrophages from 1999 to 2023, as illustrated in Figure 2.

Over a span of 25 years, a total of 1499 articles were published. The publication output experienced a gradual increase between 1999 and 2017, followed by a notable surge starting in 2018. The period from 2018 to 2023 accounted for 52.37% of the total publications.

The publication output can be categorized into three distinct phases: an initial phase from 1999 to 2010 marked by a relatively stagnant trend with gradual growth resulting in an annual publication output of less than 40 articles; a subsequent phase from 2011 to 2017 characterized by consistent annual increases in publication output, albeit at a modest rate over 7 years; and a final phase from 2018 to 2023 distinguished by a significant surge in publication output, exceeding 110 articles per year. The apex was attained in 2022, followed by a marginal decline in 2023, potentially due to certain articles not yet being incorporated into the database.

### Country and Institutional Analysis

To compare research contributions more effectively, we have combined England, Scotland, Wales, and Northern Ireland into the “United Kingdom,” to make statistical analysis more in line with international practices. The top 10 countries in terms of publication output are as follows: China, South Korea, the United States, Japan, Germany, the United Kingdom, Italy, India, Spain, and Australia. China dominates the publication landscape with a significantly higher output (n = 489) than other countries. Although South Korea (n = 238) and the United States (n = 237) have similar publication counts, the United States leads in citations with 27,464, surpassing China (8405) and South Korea (4450). The number of citations in this section represents the total number of times articles published by a country have been cited. Japan ranks fourth in terms of publication output with 146 publications, yet it holds the second position in citations with 5658 (Table 1). This suggests that a significant portion of macrophage research pertaining to osteoporosis is focused in these countries, indicating a notable concentration of research endeavors.

**TABLE 1:** Top 10 countries on research of macrophages in OP.

Rank	Country	Counts	Percent	Citations
1	CHINA	489	32.62%	8405
2	SOUTH KOREA	238	15.88%	4450
3	USA	237	15.81%	27,464
4	JAPAN	146	9.74%	5658
5	GERMANY	42	2.80%	1516
6	UNITED KINGDOM	39	2.60%	3311
7	ITALY	36	2.40%	4119
8	INDIA	24	1.60%	687
9	SPAIN	23	1.53%	595
10	AUSTRALIA	22	1.47%	1124

**FIGURE 3:**
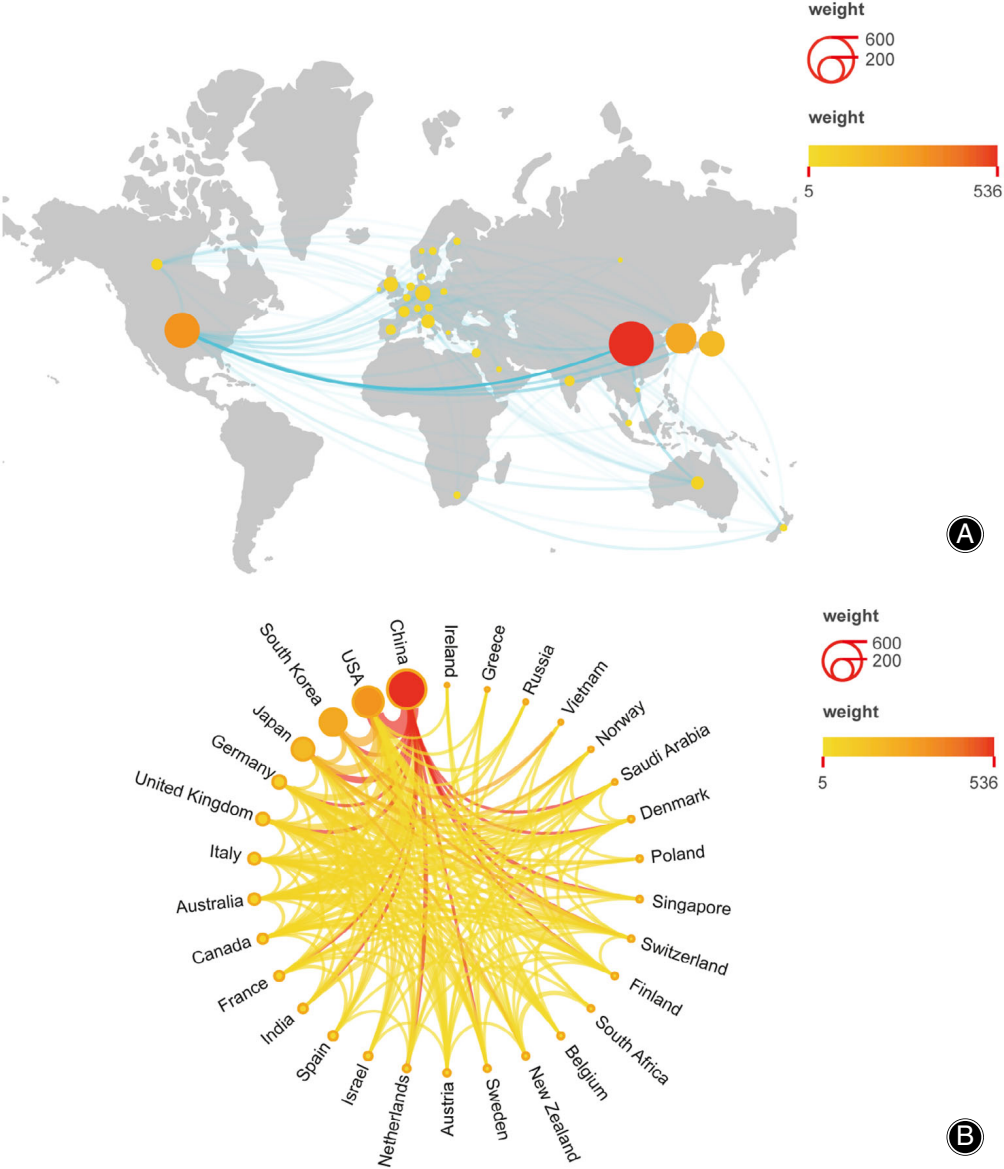
The geographical distribution (A) and visualization of countries (B) on research of macrophages in OP.

Using the Scimago Graphica software, a map network co‐occurrence analysis was conducted to examine national publication output and collaboration relationships. The findings demonstrate geographical disparities, as depicted in Figure 3A, with a notable concentration in East Asia, North America, Western Europe, and Oceania.

The presence of collaborative endeavors is apparent across various countries and regions, with a notable emphasis on the close connection between the United States and China within the collaboration network. Furthermore, positive collaborative relationships are observed between the United States and Japan, South Korea, Germany, and the United Kingdom, as well as between China and Australia and Japan. Figure 3B provides a visual representation of the top 30 countries and regions in terms of publication output. Greater circle size signifies increased publication output, while a deeper shade of red signifies more extensive collaboration with other nations. The thickness and hue of the connecting lines between countries reflect the level of intensity in research collaboration.

**FIGURE 4:**
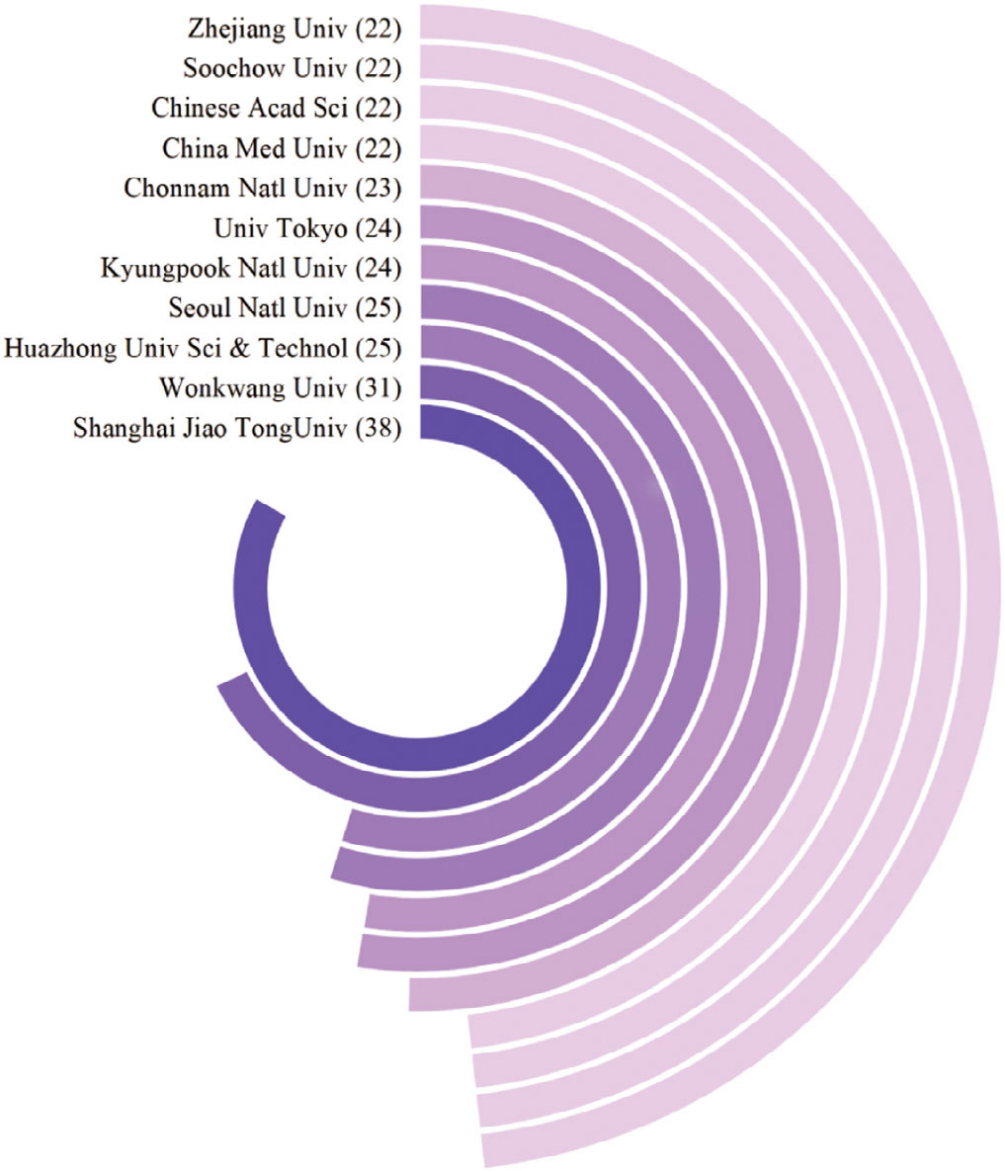
Circular distribution map of the top 10 institutions of macrophages in OP.

Table 2 displays the top 10 institutions ranked by publication output, with Shanghai Jiao Tong University leading with 38 publications, followed by Yonsei University with 31 publications in the second position. Huazhong University of Science and Technology and Seoul National University are tied for the third position, each with 25 publications. Notably, all the top 10 institutions are universities, and due to cases where the publication counts are the same, 11 institutions are presented in the table. The variations in publication productivity among the leading academic institutions are clearly demonstrated in Figure 4. Specifically, six universities in China, four in South Korea, and one in Japan, all situated in East Asia, are prominent among the major publishing institutions. Furthermore, a network visualization graph was utilized to depict collaborative relationships between institutions, focusing on 55 entities with a publication count exceeding 10.

**TABLE 2:** Top 10 institutions on research of macrophages in OP.

Rank	Counts	Counts	Citations
1	Shanghai Jiao Tong Univ	38	751
2	Wonkwang Univ	31	642
3	Huazhong Univ Sci & Technol	25	414
4	Seoul Natl Univ	25	920
5	Kyungpook Natl Univ	24	444
6	Univ Tokyo	24	1472
7	Chonnam Natl Univ	23	242
8	China Med Univ	22	408
9	Chinese Acad Sci	22	382
10	Soochow Univ	22	431
10	Zhejiang Univ	22	167

**FIGURE 5:**
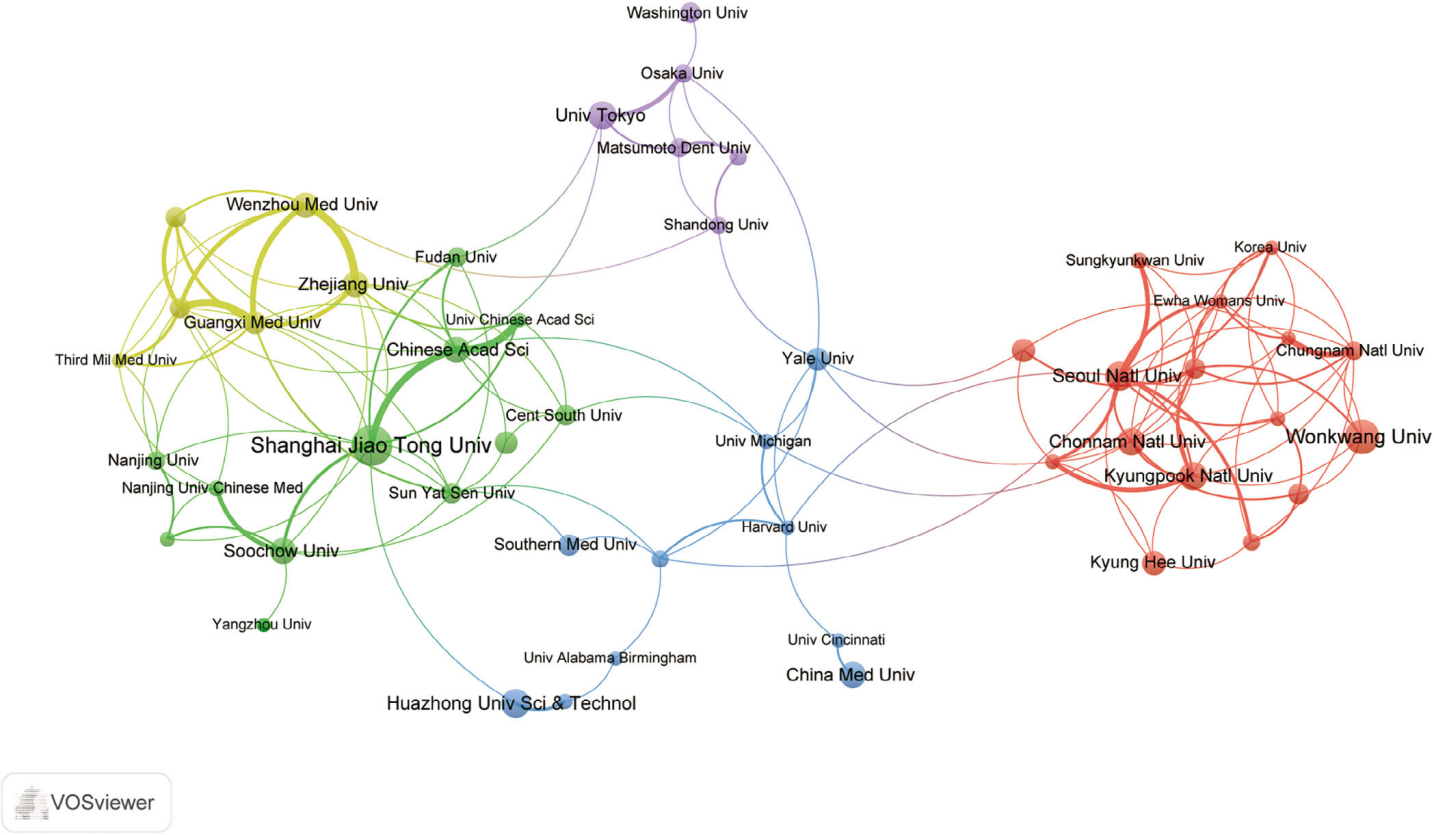
The visualization of institutions on research of macrophages in OP.

The graph in Figure 5 illustrates the number of publications and collaboration relationships for each institution. Various clusters are represented by different colors, with five distinct clusters showcasing the strength of collaboration. Shanghai Jiao Tong University, which exhibits the highest publication output, demonstrates close connections with Fudan University, the Chinese Academy of Sciences, and University of Chinese Academy of Sciences. Furthermore, significant partnerships have been established between Zhejiang University and Wenzhou Medical University, Guangxi Medical University and the University of Western Australia, as well as the University of Tokyo and Osaka Prefecture University. The data presented in the graph illustrates that a majority of collaborations are confined within a single country or among a select few institutions, indicating a lack of extensive cross‐national collaboration between academic institutions.

### Journals and Co‐Cited Journals

**FIGURE 6:**
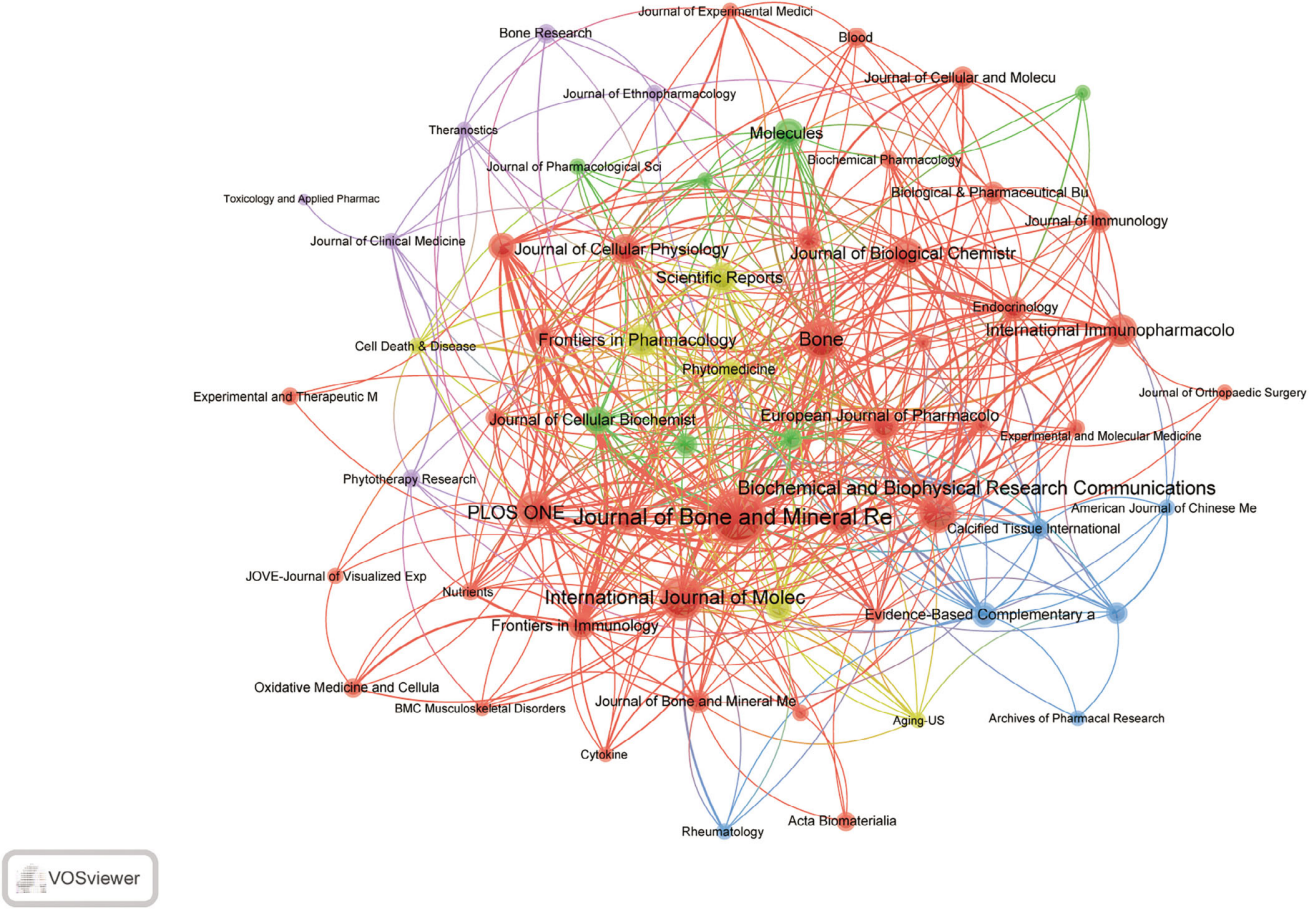
Visualization of journals on research of macrophages in OP.

**FIGURE 7:**
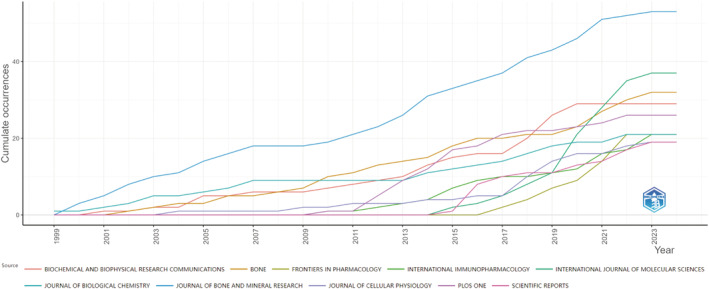
Trend of annual publication volume for the top 10 journals of macrophages in OP.

The statistical analysis of academic journals focused on macrophage research in the context of osteoporosis offers valuable insights into the scholarly influence of these studies. A comprehensive review of 534 publications in this area reveals that the *Journal of Bone and Mineral Research* has produced the largest number of articles (n = 53) and has received the highest number of citations (3365).Following are *International Journal of Molecular Sciences* (n = 37, citations = 619), *Bone* (n = 32, citations = 2220), *Biochemical and Biophysical Research Communications* (n = 29, citations = 1170), and *PLoS One* (n = 26, citations = 930). The top 20 publications exhibit a publication count of 13 or higher, with eight journals classified within the Journal Citation Reports (JCR) Q1 category and nine within the Q2 category. The highest impact factor journal is *Frontiers in Immunology* (IF = 7.3, Q1), followed by *Journal of Bone and Mineral Research* (IF = 6.2, Q1). The publication with the highest average citation count is the *Journal of Biological Chemistry*, publishing 21 articles with 2633 citations, averaging over 125 citations per article, indicating its high impact in this research field. Refer to Table 3 for more details. Subsequently, we filtered publications with five or more articles, resulting in 62 publications. A social network visualization was conducted, as shown in Figure 6. Examination of publication trends within the top 10 journals specializing in macrophage research in osteoporosis reveals a noticeable uptick in scholarly interest in this area over the past few years. Among these journals, *Journal of Bone and Mineral Research*, *International Journal of Molecular Sciences*, and *Bone* stand out as the top three publications experiencing the most rapid growth in output over the last 5 years, as illustrated in Figure 7.

**TABLE 3:** Top 20 journals for research of macrophages in OP.

Journal	Count	Citation	AVG‐Citation	IF	JCR
Journal of Bone and Mineral Research	53	3365	63.49	6.2	Q1
International Journal of Molecular Sciences	37	619	16.73	5.6	Q1
Bone	32	2220	69.38	4.1	Q2
Biochemical and Biophysical Research Communications	29	1170	40.34	3.1	Q3
PLoS One	26	930	35.77	3.7	Q2
Frontiers in Pharmacology	21	187	8.90	5.6	Q1
International Immunopharmacology	21	243	11.57	5.6	Q1
Journal of Biological Chemistry	21	2633	125.38	4.8	Q2
Journal of Cellular Physiology	19	543	28.58	5.6	Q1
Scientific Reports	19	493	25.95	4.6	Q2
European Journal of Pharmacology	18	266	14.78	5.0	Q2
Molecules	18	165	9.17	4.6	Q2
Frontiers in Endocrinology	16	150	9.38	5.2	Q1
Frontiers in Immunology	16	511	31.94	7.3	Q1
Journal of Cellular Biochemistry	16	596	37.25	4.0	Q2
Endocrinology	14	1018	72.71	4.9	Q2
FASEB Journal	14	367	26.21	4.8	Q1
Osteoporosis International	14	689	49.21	4.0	Q2
Evidence‐based Complementary And Alternative Medicine	13	112	8.62	2.6	Q4
Molecular Medicine Reports	13	144	11.08	3.4	Q3

**FIGURE 8:**
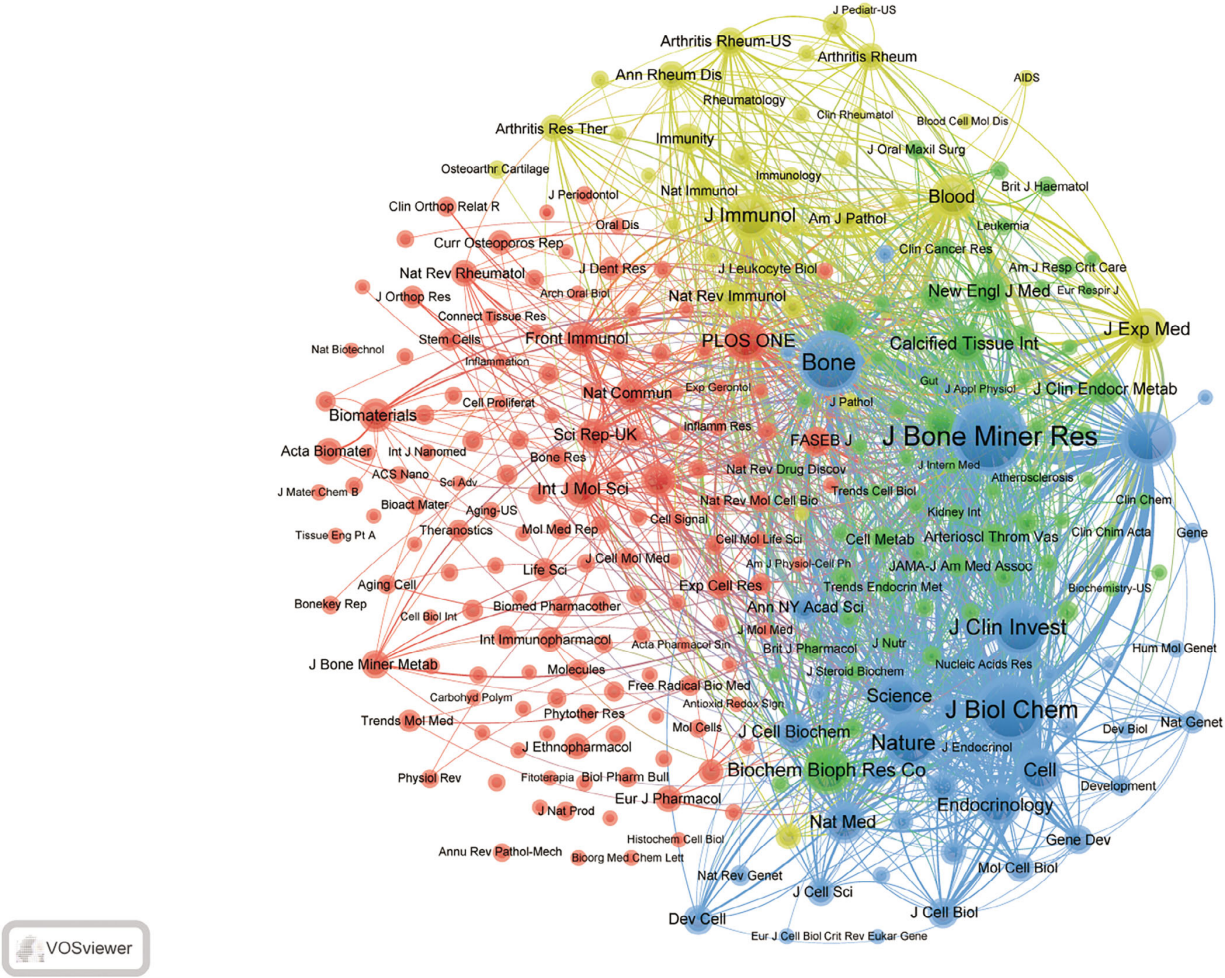
Visualization of cocited journals on research of macrophages in OP.

The co‐cited literature originates from a total of 5541 journals. The visualization of journals with more than 50 co‐citations, as depicted in Figure 8, illustrates robust co‐citation connections among *Journal of Bone and Mineral Research*, *Journal of Biological Chemistry*, and *Nature*. Publications in these journals possess substantial foundational knowledge relevance for macrophage research within the osteoporosis field, indicating the fundamental knowledge framework and evolving trends in this specific research area.

### Authors and Co‐Cited Authors

**FIGURE 9:**
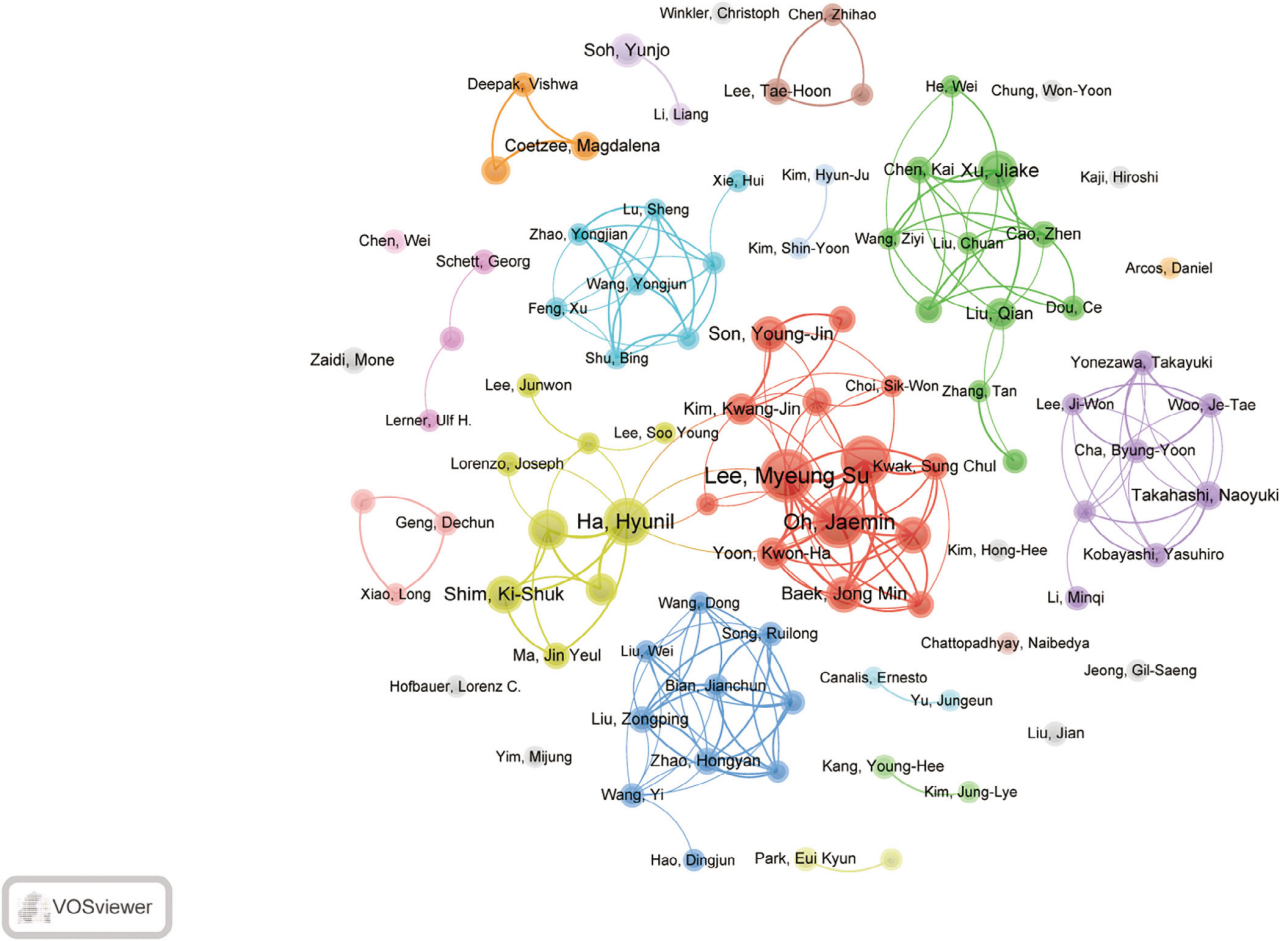
The visualization of authors on research of macrophages in OP.

Within this particular field of study, a collective total of 8744 authors have made contributions, with Lee Myeung Su (n = 20), Oh Jaemin (n = 19), and Kim Ju‐young (n = 18) ranking as the top three most prolific authors in this area of research. Price's Law can be applied to assess the uniformity of author distribution in scientific literature and to identify influential core authors. By using the formula N = 0.749 × (ηmax)^(1/2) (where ηmax denotes the publication count of the most productive author), one can determine the minimum publication count N required for an author to be classified as a core author. In this specific research field, the calculated value of N = 3.35 suggests that authors with four or more publications are considered core authors. The findings from the VOSviewer analysis indicate the presence of 174 core authors who have collectively produced 964 publications since 1999, representing 64.3% of the total publication output. This highlights the substantial contribution of core authors to the overall publication output. The top 10 authors, ranked by publication count, are presented in Table 4. Utilizing VOSviewer, a collaborative network map of authors with five or more articles was constructed, as illustrated in Figure 9. Various colors denote unique clusters, indicating that authors within the same cluster exhibit stronger collaborative ties, leading to the formation of relatively stable collaborative groups. Interactions between distinct clusters are constrained, implying limited collaboration among authors, yet highlighting substantial potential for collaboration between teams.

**TABLE 4:** Top 10 authors and co‐cited authors on research of macrophages in OP.

Authors	Count	Co‐Cited Authors	Citations
Lee Myeung Su	20	Takayanagi	581
Oh Jaemin	19	Teitelbaum	377
Kim Ju Koung	18	Boyle	342
Ha Hyunil	17	Asagiri	220
Kim Taesoo	13	Boyce	194
Xu Jiake	13	Suda	177
Shim Ki Shuk	12	Lacey	166
Baek Jong Min	11	Kong	160
Cheon Yoon Hee	11	Yasuda	158
Son Young Jin	11	Khosla	147

**FIGURE 10:**
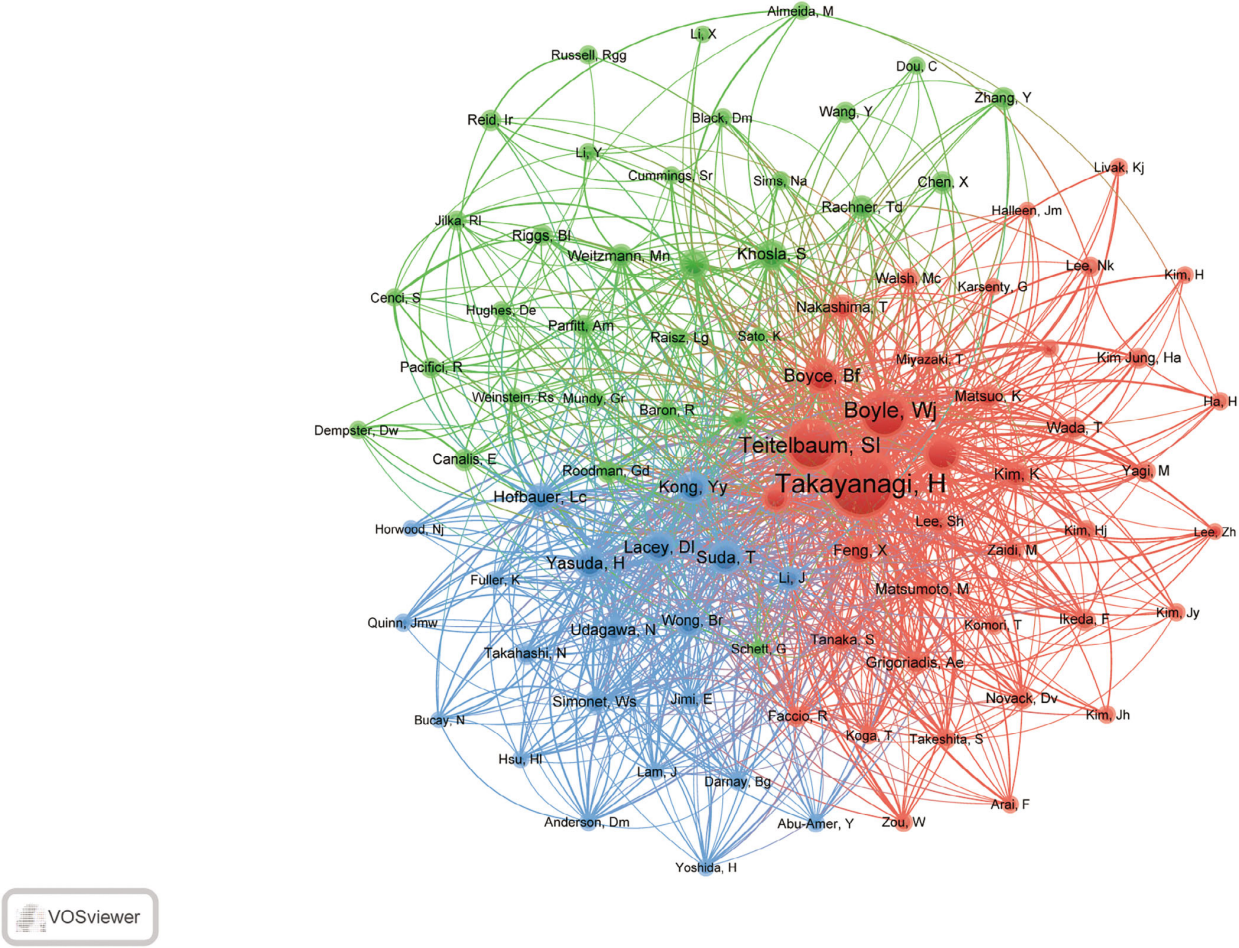
The visualization of co‐cited authors on research of macrophages in OP.

Examining co‐cited authors can provide insight into the impact of authors within a specific research field. A total of 37,713 authors were identified in the co‐cited author analysis, with 92 authors meeting the criteria of a minimum co‐citation threshold of 50. The co‐citation network diagram is depicted in Figure 10. Takayanagi is identified as the most frequently co‐cited author, with a cumulative co‐citation count of 581, followed by Teitelbaum (n = 377) and Boyle Wj (n = 342). A total of 20 authors have accrued a co‐citation count surpassing 100 (Table 4). The network diagram illustrates Takayanagi's central placement within the co‐citation network, indicating the dissemination of seminal scholarly works in the realms of osteoporosis and macrophage research.

### Co‐Cited References and Reference with Citation Bursts

**FIGURE 11:**
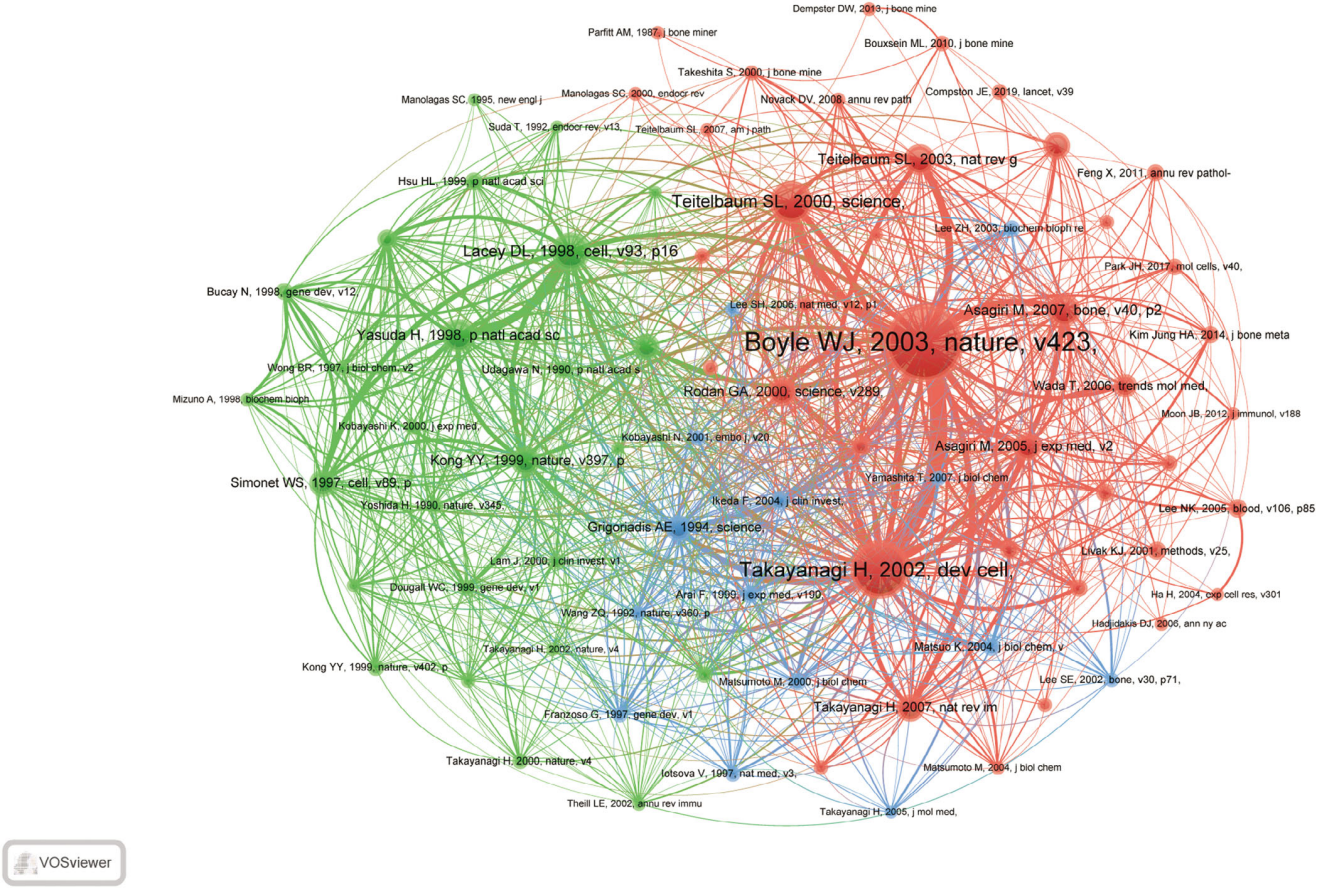
The visualization of co‐cited references on research of macrophages in OP.

The term “co‐cited literature” pertains to documents that have been referenced by multiple publications, offering valuable information on the fundamental knowledge framework within a specific research domain. Through the utilization of VOSviewer software for examination, a comprehensive total of 55,657 co‐cited documents were detected within the previous quarter‐century. Among these, eight documents garnered co‐citation counts surpassing 100, signifying their notable recognition and scholarly significance. The top 20 co‐cited documents are delineated in Table 5. Utilizing a minimum co‐citation threshold of 30, a total of 80 co‐cited documents were chosen for the creation of a network diagram. The diagram, depicted in Figure 11, highlights the presence of dynamic co‐citation relationships among various documents, including those authored by “Boyle WJ, 2003, nature,” “Takayanagi, 2002, dev cell,” “Teitelbaum, 2000, science,” “Lacey, 1998, cell,” “Yasuda, 1998, p natl acad sci usa,” and “Kong YY, 1999, nature.”

**TABLE 5:** Top 10 co‐cited references on research of macrophages in OP.

Rank	Cited reference	Citations
1	Boyle WJ, 2003, nature, v423, p337, doi10.1038/nature01658	342
2	Takayanagi H, 2002, dev cell, v3, p889, doi10.1016/s1534‐5807(02)00369‐6	244
3	Teitelbaum SL, 2000, science, v289, p1504, doi10.1126/science.289.5484.1504	169
4	Lacey DL, 1998, cell, v93, p165, doi10.1016/s0092‐8674(00)81569‐x	149
5	Yasuda H, 1998, p natl acad sci usa, v95, p3597, doi10.1073/pnas.95.7.3597	123
6	Asagiri M, 2007, bone, v40, p251, doi10.1016/j.bone.2006.09.023	118
7	Teitelbaum SL, 2003, natrevgenet, v4, p638, doi10.1038/nrg1122	113
8	Kong YY, 1999, nature, v397, p315, doi10.1038/16852	102
9	Rodan GA, 2000, science, v289, p1508, doi10.1126/science.289.5484.1508	99
10	Asagiri M, 2005, jexpmed, v202, p1261, doi10.1084/jem.20051150	97
11	Takayanagi H, 2007, natrevimmunol, v7, p292, doi10.1038/nri2062	95
12	Rachner TD, 2011, lancet, v377, p1276, doi10.1016/s0140‐6736(10)62349‐5	93
13	Timonet WS, 1997, cell, v89, p309, doi10.1016/s0092‐8674(00)80209‐3	92
14	Suda T, 1999, endocrrev, v20, p345, doi10.1210/er.20.3.345	90
15	Grigoriadis AE, 1994, science, v266, p443, doi10.1126/science.7939685	89
16	Wada T, 2006, trendsmolmed, v12, p17, doi10.1016/j.molmed.2005.11.007	78
17	Anderson DM, 1997, nature, v390, p175, doi10.1038/36593	58
17	Tagi M, 2005, jexpmed, v202, p345, doi10.1084/jem.20050645	58
19	Takayanagi H, 2007, annnyacadsci, v1116, p227, doi10.1196/annals.1402.071	57
20	Lee NK, 2005, blood, v106, p852, doi10.1182/blood‐2004‐09‐3662	56

**FIGURE 12:**
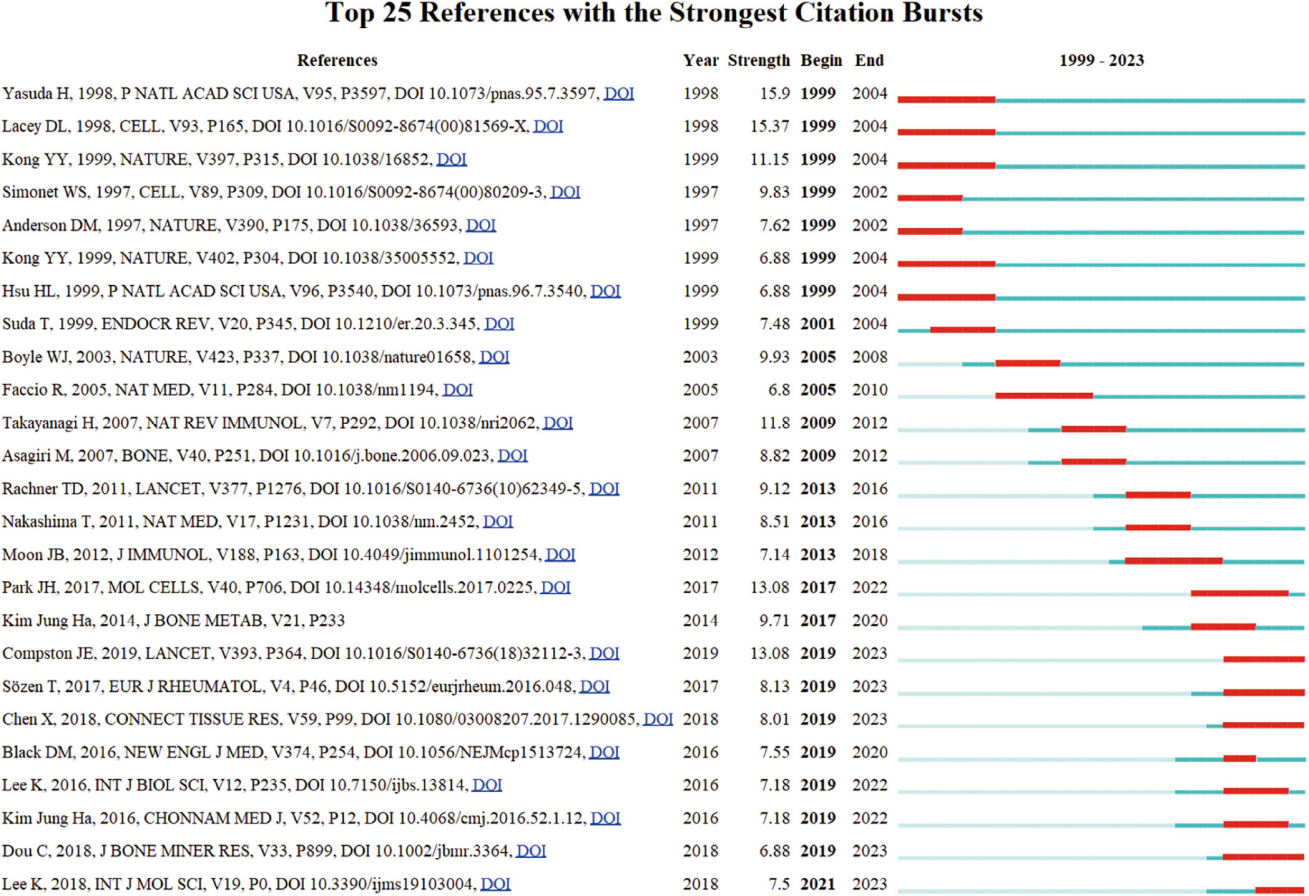
Top 25 references with strong citation bursts. Tips: A red bar indicates high citations in that year.

Furthermore, a citation burst analysis was conducted on the literature referenced in the past 25 years, utilizing Citespace software to identify and visualize the top 25 literature bursts, as depicted in Figure 12. Citation bursts indicate the scholarly works that have been extensively cited within a specific timeframe, showcasing shifts in research focus. The data presented in Figure 12 reveals that the burst values range from 6.8 to 15.9, with most bursts lasting between 3 to 5 years. The earliest burst detection occurred in 1999, with the paper “Osteoclast differentiation factor is a ligand for osteoprotegerin/osteoclastogenesis‐inhibitory factor and is identical to TRANCE/RANKL” by Yasuda exhibiting the strongest citation burst.

### Hotspots and Frontiers

**FIGURE 13:**
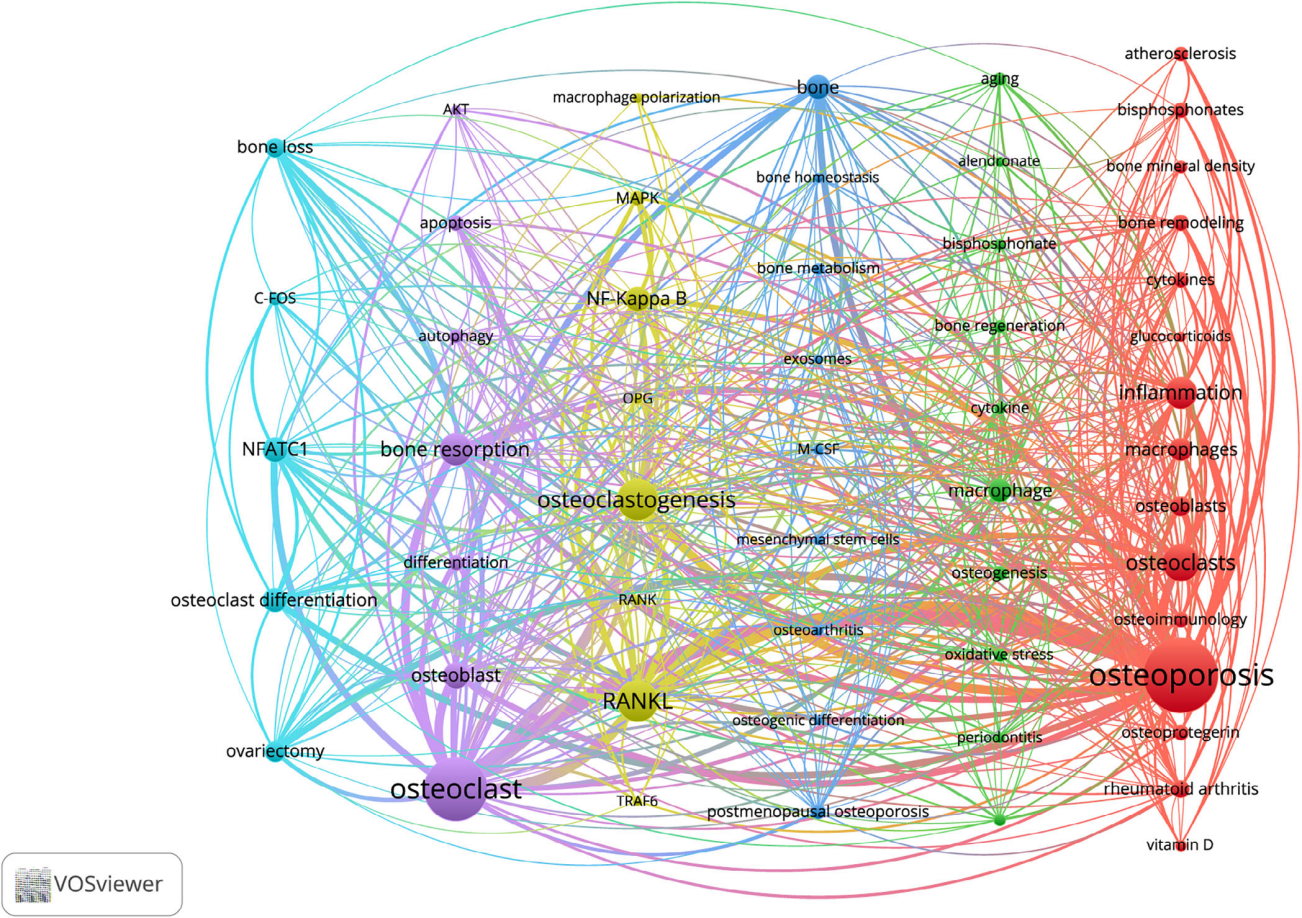
Keyword cluster analysis on research of macrophages in OP.

After conducting VOSviewer analysis, a total of 2925 author keywords were identified. The top 10 keywords by frequency are presented in Table 6, with terms such as osteoporosis, osteoclast, osteoclastogenesis, and RANKL showing frequencies exceeding 100. After applying a keyword filtering process with a minimum frequency threshold of 10, a total of 54 keywords were identified. This was followed by the generation of a keyword co‐occurrence network diagram (Figure 13), which illustrated significant associations between terms such as osteoporosis, osteoclast, bone resorption, and osteoclast formation. The 54 keywords are categorized into six clusters, as depicted in the figure. Cluster one (blue) encompasses terms like bone loss, c‐fos, nfatc1, osteoclast differentiation, and ovariectomy. Cluster two (purple) includes AKT, apoptosis, autophagy, bone resorption, differentiation, osteoblast, and osteoclast, summarizing the mechanistic aspects. Cluster three (yellow) involves macrophage polarization, MAPK, nf‐kappa b, RANKL, osteoclastogenesis, primarily focusing on signal pathway research. Cluster four (deep blue) covers bone, bone homeostasis, bone metabolism, mesenchymal stem cells, exosomes, and m‐csf, related to aspects of bone loss. Cluster five (green) features alendronate sodium, bisphosphonates, bone regeneration, macrophage, and osteogenesis, associated with the treatment of osteoporosis. Finally, cluster six (red) incorporates osteoporosis, bone mineral density, inflammation, and osteoimmunology, summarizing keywords related to the pathogenesis of osteoporosis.

**TABLE 6:** Top 10 keywords on research of macrophages in OP.

Rank	Keyword	Occurrences
1	Osteoporosis	444
2	Osteoclast	334
3	Osteoclastogenesis	151
3	RANKL	151
5	Osteoclasts	126
6	Bone Resorption	96
7	Inflammation	94
8	Osteoblast	66
9	NF‐Kappa B	59
9	NFATC1	59

**FIGURE 14:**
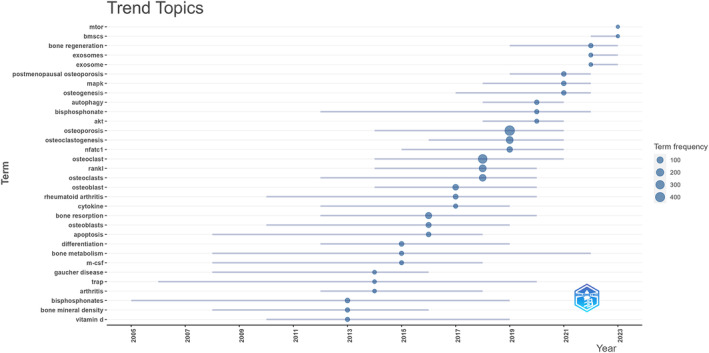
Trend topic analysis on research of macrophages in OP.

Furthermore, a keyword topic trend analysis was performed utilizing the Bibliometrix R package, as depicted in Figure 14. The trends in keyword research indicate the primary focus of studies during a specific timeframe, offering valuable insights into the advancement of macrophage research within the realm of osteoporosis. Prior to 2013, keyword analysis primarily focused on topics related to bisphosphonates, bone density, and vitamin D, highlighting the therapeutic aspects of osteoporosis. By around 2019, there was a noticeable shift in predominant keywords towards mechanisms involving osteoclast formation, osteoclast differentiation factors, and the nfatc1 gene. In recent years, there has been a gradual transition in research trends towards topics such as bone marrow mesenchymal stem cells, the mTOR signaling pathway, and bone regeneration.

## Discussion

To the best of our knowledge, this study represents the inaugural utilization of bibliometrics to conduct a comprehensive examination and projection of trends and advancements within the realm of macrophage research in the context of osteoporosis. Over the course of the last quarter‐century, research pertaining to macrophages in the osteoporosis field has demonstrated consistent advancement. Through a systematic review and analysis of pertinent literature, this study facilitates an understanding of the historical evolution and research trajectory within this domain. Additionally, it affords insights into potential disciplinary frontiers, elucidating the developmental progression, current status, and potential future directions.

An analysis of the WOSCC database reveals a notable upward trend in the number of publications focusing on macrophages in the context of osteoporosis over the last quarter‐century. Broadly categorized, this progression unfolds in three distinct phases: a period of gradual growth (1999–2010), a sustained upward trend (2011–2017), and a phase of rapid development (2018–2023). This pattern indicates the increasing importance of macrophage research in the study of osteoporosis. Currently, China has established itself as the primary contributor to publications in this particular field, representing 32.62% of the worldwide total, highlighting China's prominent role in advancing research in this area. Despite ranking third in publication volume with 237 papers, the U.S. high citation count indicates the significant influence of its research contributions. The top 10 institutions in terms of publication volume are primarily situated in East Asia, with six universities hailing from China. This distribution underscores the significant contribution of university research departments in the field. Particularly noteworthy is the substantial number of publications from Shanghai Jiao Tong University in China and Yonsei University in South Korea, each exceeding 30, indicating their significant engagement in macrophage research related to osteoporosis. Examination of the research collaboration network demonstrates extensive international cooperation, particularly evident in partnerships between China and the United States. However, the level of cooperation among institutions seems to be somewhat restricted, resulting in the formation of fragmented, small‐scale collaborative groups. This indicates that the extent and intensity of collaboration may not be at its most effective level. It is recommended that stronger international exchanges and collaboration among institutions be encouraged in order to collectively enhance research on macrophages in the field of osteoporosis.

Regarding the research on osteoporosis and macrophages, the most prolific journal is the *Journal of Bone and Mineral Research* (IF = 6.2, Q1), currently considered the most popular journal in this research domain. Scholars are advised to prioritize journals with increased publication frequency in order to expeditiously access the latest advancements in macrophage research within the realm of osteoporosis. This approach is conducive to staying abreast of developments in the research and aids in selecting suitable journals for submission, thereby avoiding any compromise on the timeliness of research outcomes. Concerning authors, Lee Myeung Su leads with 20 publications, closely followed by Oh Jaemin (n = 19) and Kim Ju Koung (n = 18). Through an analysis of the collaborative network, the collective efforts of these three researchers have resulted in the co‐authorship of numerous publications, thereby solidifying the formation of a cohesive research team.

In 2009, Lee et al.^26^ employed a mouse model to illustrate the inhibitory effect of alendronate on RANKL‐mediated differentiation of bone macrophages into osteoclasts in a dose‐dependent manner, providing insight into the therapeutic mechanism of alendronate in treating osteoporosis. Additionally, Lee et al. conducted in vitro experiments in the same year,^27^ demonstrating that the pan‐JAK inhibitor pyridone 6 suppresses the expression of c‐Fos and NFAT‐c1 in bone macrophages, leading to the inhibition of osteoclast differentiation and bone resorption activity in mature osteoclasts. The researchers hypothesized that inhibitors targeting the JAK signaling pathway could potentially be utilized in the management of osteoporosis.

The primary focus of the study is on the inhibition of macrophage differentiation into osteoclasts through the suppression of RANKL signal transduction, resulting in the inhibition of bone resorption. RANKL, a key molecule linking the skeletal and immune systems, plays a crucial role in this process, and effective blocking of RANK leads to the inhibition of bone resorption.^28^

Takayanagi is the most frequently co‐cited author, with a co‐citation count of 581. In a 2002 publication in *Nature*,^29^ Takayanagi elucidated the mechanism by which IFN‐β inhibits osteoclast differentiation by interfering with RANKL‐induced c‐Fos expression. Mice lacking IFN‐β signaling exhibited severe bone loss and increased osteoclastogenesis. Subsequently, in 2005, Takayanagi et al.^30^ found that immunosuppressants, while reducing NFAT protein activity and blocking osteoclast differentiation, also inhibited bone formation by osteoblasts. This discovery offers valuable insights for the management of osteoporosis following organ transplantation. Takayanagi's study focuses on the inhibition of osteoclast differentiation, investigation of diverse proteins, signaling pathways, and transduction factors, and establishes a theoretical foundation for comprehending the regulatory mechanisms of macrophages in osteoporosis.

Citation analysis offers a comprehensive understanding of the evolution of macrophage research within the osteoporosis field, facilitating the anticipation of forthcoming research directions and advancements. By examining co‐cited literature, valuable insights into the fundamental elements of macrophage research in osteoporosis are obtained. The study by Boyle et al.^31^ is the most commonly co‐cited research, with 342 co‐citations. It provided insights into the activation of the RANKL signaling pathway in osteoclast formation, the mechanisms of bone resorption, and the hormonal effects on bone structure. This seminal work established the groundwork for further molecular studies on macrophage‐derived osteoclasts. The second‐ranked study, by Takayanagi et al. in 2002 (co‐citations = 244), has been discussed previously. The third‐ranked study, by Teitelbaum (co‐citations = 169) in 2000,^32^ focused on the role of osteoclasts in bone resorption in osteoporosis and remains a pivotal research topic in the field.

References with citation bursts indicate emerging trends in a particular research field, as these publications are commonly cited by researchers within a specific timeframe. The article^33^ with the highest citation burst intensity, authored by Yasuda. in 1998, revealed the role of osteoprotegerin ligand (OPGL) in promoting T‐cell growth and dendritic cell function, thereby not only regulating osteoclastogenesis but also impacting the immune system. Lacey et al.^34^ conducted in vivo and in vitro experiments that revealed the ability of OPGL to bind to a distinct hematopoietic progenitor cell, thereby promoting osteoclast differentiation. Kong et al.^35^ subsequently demonstrated in animal studies in 1999 that OPGL facilitates osteoclast generation and is essential for lymph node formation and lymphocyte development. These seminal studies established the groundwork for the field of bone immunology. In 2007, Takayanagi^36^ conducted a comprehensive review of the literature on bone immunology, establishing a theoretical foundation for the field. A decade later, Park and colleagues^37^ synthesized the regulatory mechanisms of the RANK signaling pathway in osteoclastogenesis, raising concerns about potential negative impacts on immune organs from targeted therapies. Analysis of highly cited articles indicates that the exploration of various signaling pathways in osteoclast metabolism continues to be a prominent focus in contemporary research.^9^, ^38^

Research on the role of macrophages in the pathogenesis of osteoporosis has been ongoing. Given the importance of macrophages as a key source of osteoclasts, inhibiting this process and mitigating excessive bone resorption have emerged as critical areas of investigation. Recent studies have focused on exosomes, bone regeneration, and mTOR signaling pathways in macrophage research within the context of osteoporosis.

Since 2022, there have been a total of 17 studies pertaining to exosomes, five studies focusing on BMCSs, and a cumulative of five studies specifically addressing mTOR. Among these, five studies on mTOR were published in 2023. The complex interplay among macrophage polarization, BMCSs, and exosomes has recently garnered significant attention in the academic literature. Macrophages have the ability to adopt different polarization states in diverse microenvironments, with M2 macrophages playing a crucial role in the differentiation and activation of osteogenic precursors,^39^ such as BMSCs, thereby facilitating bone formation. Exosomes, membranous vesicles facilitating intercellular signaling, facilitate the transfer of biomacromolecules to receptors, thereby modulating various biological processes. Their implication in the impairment of osteogenic cell differentiation is pivotal in the development of osteoporosis.^40^ Research suggests^41^ that exosomes derived from M2 macrophages can modulate gene expression and protein synthesis by transporting miRNA, long non‐coding RNA, and functional proteins. Liu et al.^42^ conducted in vitro experiments that revealed exosomal miR‐486‐5p derived from M2 macrophages enhances the osteogenic potential of bone marrow mesenchymal stem cells while suppressing their adipogenic differentiation. Additionally, animal studies demonstrated that miR‐486‐5p mitigates bone loss in ovariectomized mice. Xiong et al.^43^ discovered that M2 macrophages derived from mouse bone marrow macrophages exhibited an enrichment of miR‐5106. Furthermore, they observed that exosomes containing miR‐5106 (M2D‐Exo) facilitated the osteogenic differentiation of bone marrow mesenchymal stem cells by downregulating the expression of osteogenic‐related genes SIK2 and SIK3, thereby expediting bone fracture healing in an in vivo setting. Significantly, Ma et al.^44^ found that the fusion of exosomes derived from bone marrow mesenchymal stem cells (BMSCs‐Exos) and exosomes from M2 macrophages (M2‐Exos) into M2‐BMSCs‐Exos combines the beneficial properties of both sources, resulting in a targeted inhibitory effect on bone resorption. This discovery represents a significant advancement in the development of targeted formulations for osteoporosis.

mTOR, a constituent of the phosphoinositide 3‐kinase‐related kinase (PIKK) protein family, orchestrates both upstream and downstream signal transduction pathways to govern a multitude of cellular processes, such as energy metabolism, protein biosynthesis, autophagy, cellular growth, and proliferation.^45^ Its involvement is intricately linked with the pathogenesis of numerous diseases, including cancer, type 2 diabetes, obesity, neurodegenerative disorders, and aging.^46^

The process of aging is closely associated with macrophage dysfunction and the resulting inflammation induced by these immune cells. As individuals grow older, there is a notable increase in the bone resorption activity of macrophage lineage cells, such as osteoclasts and their precursors,^47^ with the mTOR pathway playing a significant role in the aging process.

Research^48^ has shown that the downregulation and inactivation of FOXO1 signaling in aging macrophages leads to decreased REDD1 expression and increased mTOR phosphorylation levels, exacerbating bone loss and macrophage aging. Concurrently, Hiraiwa et al.^49^ have illustrated that mTORC1 impacts bone homeostasis through diverse signaling pathways, exhibiting bidirectional effects under various regulatory factors. This highlights the potential significance of mTORC1 as a critical target for the treatment of osteoporosis.

## Advantages and Limitations

This study demonstrates multiple strengths. Firstly, it is the first known application of bibliometrics to systematically analyze research on macrophages in the context of osteoporosis, providing a valuable resource for scholars interested in this area. Secondly, the bibliometric tools employed in this study (VOSviewer, Citespeace, Bibliometrix R package) have been extensively utilized in various bibliometric studies, highlighting the credibility and validity of our statistical analysis findings. Finally, the bibliometric analysis allows for a more thorough examination of disciplinary focal points, offering valuable insights into current advancements.

Nevertheless, our study has certain limitations: (1) we exclusively searched the WoSCC database, potentially leading to literature omissions and incomplete data; (2) non‐English literature was not included in our analysis; (3) due to the publication cycle and inherent delays in indexing, a lag in the inclusion of literature and citations is an existing limitation in bibliometric studies. Despite these limitations, our study effectively describes the global trends in macrophage research within the osteoporosis field.

## Conclusion

In summary, this research employed literature sourced from the Web of Science database and employed various visualization tools, including VOSviewer, CiteSpace, Scimago Graphica, Bibliometrix R package, and Pajek software, to examine trends and focal areas in macrophage research within the osteoporosis domain. Statistical analyses were performed on publication volume, countries, institutions, authors, journals, references, and keywords, with the objective of offering researchers a more comprehensive insight into the evolutionary path and research priorities in this particular field. Moreover, the research indicates that investigating the interplay between extracellular vesicles originating from macrophages and bone marrow mesenchymal stem cells, as well as the influence of the mTOR signaling pathway on macrophages, has substantial promise and relevance for further exploration in the field of osteoporosis.

## Conflict of Interest Statement

The authors declare no conflicts of interest in the research.

## Author Contributions

All authors made a significant contribution to the work reported and agreed to be accountable for all aspects of the work. J.H.F., L.X.Z., and L.G. designed the experiments. L.H.Z., J.K. and R.Y.F. collected and organized the data. J.H.F. and L.H.Z. visualized the data. J.H.F. and L.X.Z. prepared the initial draft of the manuscript. L.G. gave critical feedback during the study or during the submission of the manuscript. All authors provided final approval of the version to be submitted and agreed on the journal for publication.

## Data Availability

The study includes the original contributions, which are detailed in the article and supplementary material. For additional information, inquiries may be directed to the corresponding author.
